# High BMP7 expression is associated with poor prognosis in ovarian cancer

**DOI:** 10.1111/jcmm.17951

**Published:** 2023-09-08

**Authors:** Richa Vasan, Jahnavi Yadav, Radhika Aiyappa‐Maudsley, Suha Deen, Sarah J. Storr, Stewart G. Martin

**Affiliations:** ^1^ Nottingham Breast Cancer Research Centre, School of Medicine University of Nottingham Biodiscovery Institute Nottingham UK; ^2^ Department of Pathology, Queen's Medical Centre Nottingham University Hospitals NHS Trust Nottingham UK; ^3^ Present address: School of Cancer Sciences University of Glasgow Glasgow UK; ^4^ Present address: School of medicine University of Leeds Leeds UK

**Keywords:** BMP7 protein, ovarian cancer, prognosis, survival

## Abstract

Bone Morphogenetic Protein 7 (BMP7) is an extracellular signalling protein that belongs to the transforming growth factor‐β (TGF‐ β) superfamily. Previous transcriptomic data suggested that BMP7 expression may be disrupted in ovarian carcinoma and may play an important role in the aggressiveness of the disease. However, the protein expression in patient tumours has not been well studied. The current study aimed to assess BMP7 protein expression in a large cohort of ovarian carcinoma patient tumour samples to establish its associations with different clinical endpoints. Ovarian carcinoma tissue samples from 575 patients who underwent surgery for different subtypes of ovarian cancer were used. BMP7 protein expression was analysed by immunohistochemistry using tissue microarray and full face tumour sections. High BMP7 expression is associated with aggressive ovarian cancer clinicopathological variables including advanced FIGO stage, high grade, residual disease and poor overall survival. Elevated cytoplasmic and nuclear BMP7 expression was significantly associated with advanced FIGO stage, high tumour grade, presence of residual tumours and high‐grade serous carcinomas (*p* = 0.001, 0.005, 0.004, <0.001 and *p* < 0.001, <0.001, 0.002, 0.001 respectively). Increased cytoplasmic and nuclear BMP7 expression was also significantly associated with an adverse overall survival (*p* = 0.001 and 0.046 respectively). The study highlights the potential of BMP7 as a prognostic tool and as a potential novel target for ovarian cancer therapies to limit disease progression.

## 1 BACKGROUND

Ovarian cancer is the deadliest gynaecological malignancy, with a 5‐year survival rate of only 42.6%,^1^ partly due to early stages often being asymptomatic. Standard treatment strategies are a combination of surgery and chemotherapeutic drugs such as carboplatin and paclitaxel. However, resistance remains a significant problem making patients more susceptible to recurrent disease which is associated with a worse prognosis.^2^ It is therefore important to understand the underlying mechanisms of disease progression and identify novel targets to inform therapeutic approaches and provide optimal treatment when diagnosed.

Bone morphogenetic proteins (BMPs) are a group of over 20 growth factor‐related proteins, belonging to the transforming growth factor‐β (TGF‐ β) superfamily.^3^ BMPs were originally identified to induce ectopic bone formation.^4^ These proteins are important morphogens that are involved in embryonic development and play an important role in activation of differential genes that lead to different cell fates during the dorsal‐ventral axis patterning.^5^ These are extracellular signalling cytokines that bind to Type I and Type II serine/threonine kinase BMP receptors (BMPRs) on cells, forming a heterotetramic complex.^6^ Type I and type II receptors act as ligand‐inducible transcription factors that induce signal transduction pathways involving small mothers against decapentaplegics (SMAD) proteins. BMP ligands bind to receptor BMPRI and BMPRII which is followed by BMPRII phosphorylation of BMPRI which in tun phosphorylates SMAD family members 1, 5 and 8. SMAD 1/5/8 form a heteromeric complex with SMAD 4. Activated SMAD complexes translocate into the nucleus and consequently bind to DNA consensus SMAD binding sites to regulate gene expression as they act as transcription factors.^7^, ^8^, ^9^ Regulation of BMP signalling is intricate, both inside and outside the cell. They also activate and interact with other signalling pathways, including the MAP kinase pathways.^10^

In human granulosa cells, BMP7 was shown to modulate ovarian steroidogenesis.^11^ BMP7 was shown to be expressed in theca cells and was seen to cause changes in the action of follicle‐stimulating hormone on progesterone and oestradiol production in the mammalian ovary.^12^ There are a limited number of studies, but aberrant expression of BMP7 has been reported in several cancers. Data, however, is conflicting as there are reports of BMP7's pleiotropic role in suppressing but also promoting tumour development and metastasis.^13^ Due to contrasting observations, it has been suggested that the role BMPs play in cancer is dependent on the type of tumour, its microenvironment and the epigenetic background of the patient.

Morrissey and colleagues found that BMP7 is expressed at higher levels in prostate cancer‐related bone and soft tissue metastasis in comparison to primary prostate cancer suggesting BMP7 signalling is associated with tumour metastasis and cancer progression.^14^ High expression of BMP7 was also shown to be associated with lymph node involvement in lung cancer^15^ suggesting its role in metastasis and EMT.

BMP7 works differently depending on the cancer type and its interactions with different receptors or molecules within the tumour microenvironment.^16^ Sun and colleagues reported that BMP7 expression knockdown in cervical cancer cells inhibited cell proliferation, migration, invasion and reversed the EMT process.^17^ Collectively, aberrant expression of BMP7 and its involvement in tumour metastasis has been suggested but requires further investigation to confirm the nature of its involvement. Furthermore, there is little known about BMP7 expression and its role in ovarian cancer.

Our group previously reported in vitro transcriptomic data that suggested that BMP7 protein may be important in the aggressiveness of ovarian cancer and, as a result, may possibly influence disease progression and overall survival of patients with ovarian cancer.^18^ The current study built upon such data by assessing BMP7 protein expression in a large cohort of ovarian carcinoma patient samples by standard immunohistochemistry approaches, using full face tumour sections and tissue microarrays.

## 2 METHODS

### 2.1 Clinical samples

Specimens were taken from patients diagnosed and treated for ovarian cancer at Nottingham University Hospitals between 1991 and 2011. Five hundred and seventy five ovarian cancer specimens were collected for the study: with 337 high grade serous, 30 low grade serous, 60 mucinous, 68 endometrioid, 53 clear cell and 16 borderline carcinomas. The median follow‐up time was 8 years, ranging from 3 years to 20 years. The overall survival time of patients ranged from 0 to 223 months with a median overall survival time of 44 months. Clinicopathological data of patients was collected and included the age, stage of the cancer, histological subtype, grade, type of treatment and survival outcomes. Data is summarized in Table S1. Grading was performed using the Shimizu‐Silverberg grading system, in which tumours are given a score from 1 to 3 according to the degree of 3 parameters—architectural pattern, nuclear pleomorphism and mitotic count. The summed scores of 3–5 are classified as grades 1, 6 and 7 are grade 2, whilst 8 and 9 are classified as grade 3.^19^ Patients were treated with adjuvant chemotherapy, with 63.6% receiving platinum‐based chemotherapy, and resistance to chemotherapy was classified according to the Gynaecologic Oncology Group (GOG) into groups of refractory, resistant or sensitive. Refractory was defined as having no response to chemotherapy, resistant was defined as indicating an initial response to chemotherapy, however, recurrence within 6 months and sensitive was defined as when there was either no recurrence or recurred after 6 months. 32.9% patients showed no recurrence, while 63.1% patients showed recurrence of the disease.

Ethical approval was obtained from the Derbyshire Ethics Committee (07/H0401/156). Reporting Recommendations for Tumour Marker Prognostic Studies (REMARK) guidelines were followed during this study.^20^

### 2.2 Tissue microarray

Construction of TMA's were described previously.^18^, ^21^, ^22^ Briefly, 0.6 mm cores from 575 ovarian donor tumour formalin fixed, paraffin embedded specimens were extracted and inserted into donor/recipient blocks—with up to 150 cores in each. The 0.6 mm cores were taken from a representative area as assessed by a specialist ovarian cancer histopathologist, using the method described previously.^18^, ^21^, ^22^ For most cases, a single core was extracted from a single patient biopsy, however, in 48 cases, 2 cores were extracted from one patient specimen and in 38 cases, 3 cores were extracted from each. Fresh sections of 4 μm were cut from each TMA block and placed on glass slides for evaluation by immunohistochemistry.

To examine distribution of protein expression and provide potential justification for the use of TMAs, full face sections of 4 μm were also cut from 11 donor tumours and placed on glass slides for evaluation by Immunohistochemistry. The full face sections were taken from tumours of varying histological subtype, grade and stages. In summary, the full face sections included 6 HGSC, 3 mucinous and 2 endometrioid cases, with 4 stage 1 tumours, 2 stage 2, 4 stage 3 and 1 stage 4 tumour.

### 2.3 Immunohistochemistry

Immunohistochemical staining was conducted using a Novolink Polymer Detection kit (Leica). Tissue slides were stained according to manufacturers' instructions and has been described previously.^23^ The tissue was briefly deparaffinized in xylene, rehydrated in ethanol followed by water. Slides were heated in a microwave for 10 min at 750 W followed by 10 min at 450 W in 0.01 mol L^−1^ sodium citrate buffer (pH 6.0) for antigen retrieval. Slides were incubated with Novolink Peroxidase Block to minimize endogenous peroxidase activity and then washed with Tris‐buffered saline (TBS), followed by incubation with Novolink Protein Block solution to ensure minimal non‐specific binding. Mouse monoclonal anti‐BMP7 (Santa Cruz Biotechnology) diluted 1:100 used as the primary antibody was incubated on tissue for overnight at 4°C. The antibody specificity was confirmed by Western blotting prior conducting Immunohistochemistry (Figure S1). TBS washes were done prior to tissue incubation with the Novolink Post‐Primary reagent for 30 min, followed by TBS washes for 5 min subsequently followed by incubation with Novolink Polymer solution. Protein expression was then visualized by staining with 3,3′‐ diaminobenzidine (DAB) and counterstained with haematoxylin. Positive and negative controls were included with each staining run. Breast tumour composite sections comprising grade 1 and 2 early‐stage invasive tumours were used as positive controls; negative controls had primary antibody omitted from each staining run.

Stained slides were scanned using a Nanozoomer Digital Pathology Scanner (Hamamatsu Photonics), and staining assessed at 200× magnification. Staining in the cytoplasm was assessed using a semi‐quantitative immunohistochemical H score, where staining intensity within tumour cells was assessed as none (0), weak (1), medium (2) or strong (3), over the percentage area of each staining intensity. Staining in the nucleus was examined in a semi‐quantitative manner, where the percentage of tumour cells that demonstrated any staining intensity was assessed. Greater than 30% of cores for each TMA were double assessed, with both assessors blinded to clinical outcome and each other's scores. The single measure ICCs between scores were 0.853 and 0.803 for cytoplasm and nuclear staining respectively, indicating a strong correlation. The X‐tile software was used to determine the cut‐points for assessments using patient overall survival to stratify the data into categorized high and low expression scores.^24^ The cut‐point scores were 100 and 75 for cytoplasmic and nuclear scoring, respectively.

### 2.4 Analyses using the TCGA PanCancer atlas dataset

The Cancer Genome Atlas (TCGA) program started as a joint effort between the National Cancer Institute (NCI) and the National Human Genome Research in 2006. This project has archived genetic mutations underlying different cancer types.^25^ The PanCancer Atlas project consists of an integrative analysis of approximately 10,000 samples from 33 forms of cancer that provides a comprehensive understanding of the molecular factors that determine different cancers in the TCGA.^26^ The ovarian carcinoma TCGA PanCancer Atlas data set was used to conduct additional analyses using mRNA expression data. The data set contains data summary from a broad sampling of ovarian serous cystadenocarcinoma patients (*n* = 585); however, data was not available for all the samples. BMP7 gene expression from this data set was used to determine links between BMP7 mRNA expression and patient survival. Median BMP7 expression data was categorized using X‐tile. A cut point of 2.94 was set and BMP7 expression above the cut‐point was categorized and high expression, while expression below the cut point was classed as low BMP7 expression.

### 2.5 Statistical analysis

Pearsons chi‐square test of association (*χ* ^*2*^) was used to examine the relationship between categorized protein expression and clinicopathological factors. The association between protein expression and survival outcomes was evaluated using survival curves generated by the Kaplan–Meier method and statistical significance determined by the Log‐rank test. In addition to these univariate analyses, multivariate survival analysis was conducted using a proportional hazards model by Cox regression analysis to estimate hazard ratios and 95% confidence intervals for overall survival. Spearman's rank correlation coefficient (Spearman's rho) test was performed to assess the correlation between the expression level of BMP7 and the calpain system proteins. Empirical classifications for interpreting the correlation strength using the Spearman's rho r value have been proposed by Cohen.^27^ Cohen suggests that 0.10–0.30 is a weak correlation, 0.30–0.50 a moderate correlation and greater than 0.50 a strong correlation. Statistical analyses were performed using SPSS 22.0 software and *p* < 0.05 were considered statistically significant.

## 3 RESULTS

### 3.1 Expression pattern of BMP7 in ovarian cancer

**FIGURE 1:**
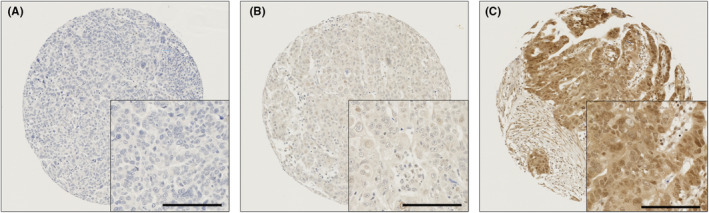
Representative photomicrographs of BMP7 expression in the cytoplasm and nucleus in ovarian carcinoma cells. Different levels of BMP7 cytoplasmic and nuclear staining (weak to intense) between adjacent tumours cells is demonstrated. Levels of expression classified as (A) negative expression (B) low expression (C) high expression in cytoplasm and nucleus, at x10 magnification with x20 magnification inset panel. Scale bar represents 100 μM.

Protein expression was studied in ovarian cancer full face tumour sections stained immunohistochemically. BMP7 was homogenously distributed throughout the tumour sections (data not shown). The homogeneity of BMP7 expression within the tumour section justified the use of tissue microarrays in this study. BMP7 expression was seen in the cytoplasm and nucleus of tumour cells with predominantly granular/diffuse staining (Figure 1).

Cytoplasmic BMP7 expression had H scores ranged from 0 to 250, with a median score of 40. X‐tile computed a H‐score cut‐point of 100, with 33% (139/424) cases demonstrating high expression. Whilst nuclear BMP7 expression had H scores ranging from 0 to 200, with a median of 42.5. X‐tile computed a H‐score cut‐point of 75, with 42% (176/424) cases demonstrating high expression. The mean cytoplasmic H score was 67.35, whilst the mean nuclear score was 60.68.

### 3.2 Correlation between cytoplasmic and nuclear BMP7

Spearman's correlation test was used to calculate the correlation between cytoplasmic and nuclear BMP7. 74.8% and 76.9% of cases showed positive cytoplasmic and nuclear staining, respectively. Cytoplasmic BMP7 expression was significantly correlated with nuclear BMP7 expression (*r* ^*2*^ = 0.790, *p* < 0.001).

### 3.3 BMP7 expression and clinicopathological factors

The relationships between BMP7 expression and clinicopathological variables were evaluated using the Pearson's chi‐square test, with results shown in Table 1. High BMP7 cytoplasmic expression associated with high tumour grade (*χ* ^*2*^ = 10.700, d.f. = 2, *p* = 0.005) and high FIGO stage (*χ* ^*2*^ = 16.762, d.f. = 3, *p* = 0.001). High cytoplasmic expression was also linked with high grade serous carcinomas (HGSC) (*χ* ^*2*^ = 37.351, d.f. = 6, *p* < 0.001) and the presence of residual disease (*χ* ^*2*^ = 11.277, d.f. = 2, *p* = 0.004). Low cytoplasmic expression associated with mucinous, endometrioid, clear cell carcinomas and low‐grade serous carcinomas (*χ* ^*2*^ = 37.351, d.f. = 6, *p* < 0.001).

**TABLE 1:** The associations between clinicopathological variables and cytoplasmic and nuclear expression of BMP7.

Variables	Cytoplasm			Nuclear		
Histological subtypes	Low	High	*p* Value	Low	High	*p* Value
High grade serous (59.7%)	147 (34.7%)	112 (26.4%)	**<0.001**	130 (30.7%)	130 (30.7%)	**0.001**
Mucinous (10.6%)	36 (8.5%)	6 (1.4%)		33 (7.8%)	8 (1.9%)	
Endometrioid (12.1%)	38 (9.0%)	12 (2.8%)		35 (8.3%)	15 (3.5%)	
Clear cell (9.4%)	42 (9.9%)	3 (0.7%)		32 (7.5%)	12 (2.8%)	
Low grade serous (5.3%)	14 (3.3%)	3 (0.7%)		11 (2.6%)	6 (1.4%)	
Boderline serous (2.7%)	7 (1.7%)	3 (0.7%)		6 (1.4%)	5 (1.2%)	
Boderline mucinous (0.2%)	1 (0.2%)	0 (0.0%)		1 (0.2%)	0 (0.0%)	
Age						
<61 (52.4%)	128 (30.3%)	62 (14.7%)	0.998	109 (25.8%)	79 (18.7%)	0.808
>61 (47.4%)	157 (37.1%)	76 (18.0%)		139 (32.9%)	96 (22.7%)	
Grade						
1 (8.5%)	27 (6.4%)	7 (1.7%)	**0.005**	21 (5.0%)	12 (2.8%)	**<0.001**
2 (16%)	50 (11.8%)	11 (2.6%)		50 (11.8%)	12 (2.8%)	
3 (75.5%)	208 (49.1%)	121 (28.5%)		177 (41.7%)	52 (35.8%)	
FIGO Stage						
1 (36.7%)	121 (57.4%)	31 (7.4%)	**0.001**	109 (26.1%)	43 (10.3%)	**<0.001**
2 (11.6%)	28 (6.7%)	17 (4.1%)		21 (5.0%)	24 (5.7%)	
3 (44.3%)	113 (27.0%)	77 (18.4%)		101 (24.2%)	89 (21.3%)	
4 (7.4%)	19 (4.5%)	12 (2.9%)		14 (3.3%)	17 (4.1%)	
Stage						
Confined tumour (I) (36.7%)	121 (28.9%)	31 (7.4%)	**<0.001**	109 (26.1%)	43 (10.3%)	**<0.001**
Tumour spread (II‐IV) (63.3%)	160 (38.2%)	60 (25.4%)		136 (32.5%)	130 (31.1%)	
Distant Metastasis						
Absent (I‐III) (92.6%)	262 (62.6%)	125 (29.9%)	**<0.001**	231 (55.3%)	56 (37.3%)	**<0.001**
Present (IV) (7.4%)	19 (4.5%)	12 (2.9%)		14 (3.3%)	17 (4.1%)	
Response to chemotherapy						
Pt resistant (17.7%)	39 (14.1%)	11 (4.0%)	0.259	32 (11.6%)	18 (6.5%)	0.632
Pt sensitive (82.3%)	159 (57.4%)	68 (24.5%)		137 (49.5%)	90 (32.5%)	
Residual disease						
No residual tumour (62.2%)	176 (49.9%)	54 (14.4%)	**0.004**	156 (41.6%)	74 (19.7%)	**0.002**
Residual tumour <2 cm (11.6%)	32 (8.5%)	12 (3.2%)		26 (6.9%)	18 (4.8%)	
Residual tumour >2 cm (26.2%)	59 (15.7%)	42 (11.2%)		48 (12.8%)	53 (14.1%)	

*Note*: Significant results (*p* < 0.05) are highlighted in bold.

High BMP7 nuclear expression also linked to the same variables as with high cytoplasmic expression, including high tumour grade (*χ* ^*2*^ = 15.876, d.f. = 2, *p* < 0.001) and high FIGO stage (*χ* ^*2*^ = 18.039, d.f. = 3, *p* < 0.001). High nuclear expression also associated with high grade serous carcinoma (*χ* ^*2*^ = 23.343, d.f. = 6, *p* = 0.001) and the presence of residual disease (*χ* ^*2*^ = 12.302 d.f. = 2, *p* = 0.002). Low nuclear expression was associated with mucinous, endometrioid, clear cell carcinomas and low‐grade serous carcinomas (*χ* ^*2*^ = 23.343, d.f. = 6, *p* = 0.001).

According to tumour stage, cases were categorized into whether they had an organ‐confined tumour (i.e. confined group: stage 1 and spread group: stage 2–4) or whether distant metastases were observed (i.e. absent group: stage 1–3 and present group: stage 4). Cytoplasmic and nuclear expression showed a significant association with organ‐confined tumours, however no significant association was found in cancers with distant metastases. High cytoplasmic and nuclear expression were both associated with ovarian cancers that has spread to other secondary organs (*χ* ^*2*^ = 16.616, d.f. = 1, *p* < 0.001 and *χ* ^*2*^ = 16.893, d.f. = 1, *p* < 0.001 respectively).

### 3.4 BMP7 expression and clinical outcomes

**FIGURE 2:**
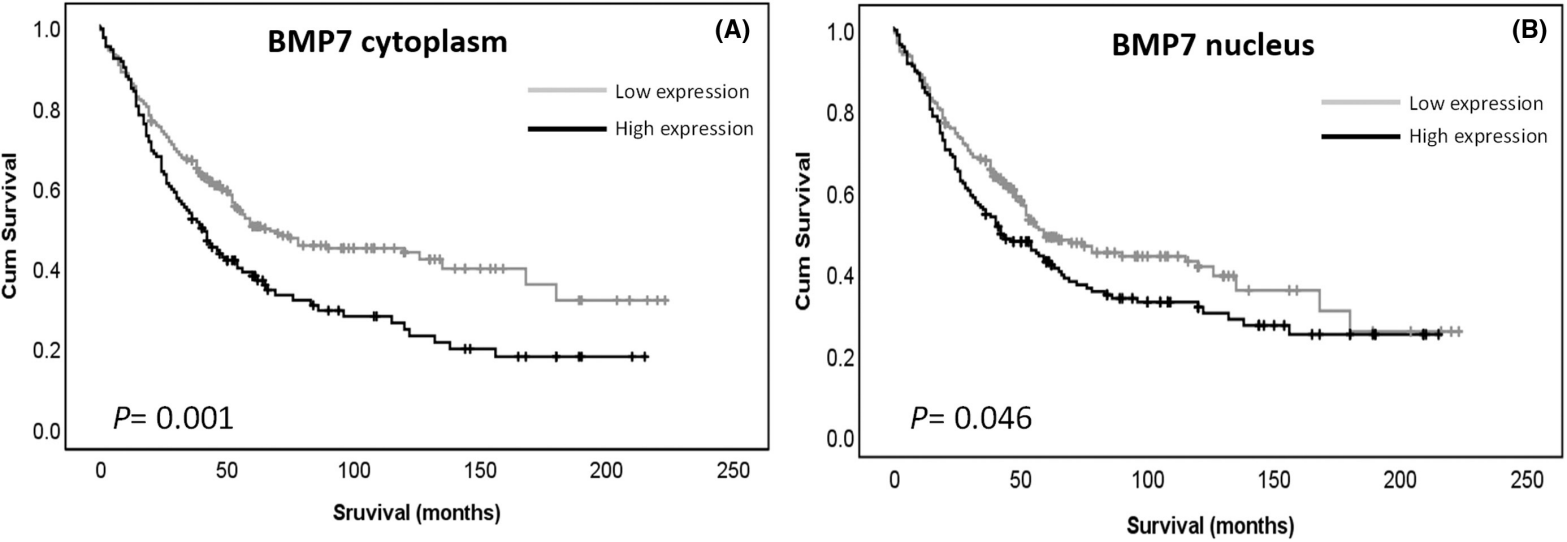
Kaplan–Meier survival curves of BMP7 cytoplasmic and nuclear expression and overall survival. (A) Survival analysis shows significantly better (*p* = 0.001) overall survival for ovarian cancer patients with tumours of low BMP7 expression in the cytoplasm compared to those with tumours of high BMP7 expression in the cytoplasm. (B) Survival analysis shows significantly better (*p* = 0.046) overall survival for ovarian cancer patients with tumours of low BMP7 expression in the nucleus compared to those with tumours of high BMP7 expression in the nucleus. Significance was determined using the log‐rank test. High expression—black line, low expression—grey line.

Kaplan–Meier survival analyses were conducted to analyse the relationship between BMP7 expression and overall survival, as well as progression‐free survival. High BMP7 cytoplasmic and high nuclear expression significantly associated with adverse overall survival (*p* = 0.001 and *p* = 0.046 respectively) (Figure 2).

No significant association was found between cytoplasmic or nuclear protein expression and progression free survival (PFS) (*p* = 0.287 and 0.506 respectively).

Using the *p‐*values from the log rank test of less than 0.001, grade, histological subtypes, age, FIGO stage, platinum sensitivity and presence of residual tumour were included in multivariate analysis. Neither cytoplasmic nor nuclear expression was found to be an independent marker of overall survival (Table S2) (*p* = 0.327 and *p* = 0.909 respectively). FIGO stage and platinum sensitivity were found to be independent markers of overall survival (*p* = 0.002 and *p* < 0.001 respectively).

### 3.5 Correlation with calpain system expression

The patient cohort used in this study was the same as that previously reported by Zhang et al., 2019, studying the expression of calpain system proteins. In vitro data obtained previously indicated the potential significance of BMP7 expression in ovarian cancer. EMT related gene expression profiling demonstrated the ovarian cancer cell line PEO4 had a 260‐352‐fold overexpression of BMP7 gene with inhibition of calpains by calpeptin treatment compared to the vehicle treated cells.^18^ To compare BMP7 expression with other potential prognostic biomarkers, protein expression of Calpastatin, calpain 1, calpain 2 and calpain 4 was compared with the cytoplasmic and nuclear expression of BMP7 using Spearman's correlation test. BMP7 cytoplasmic and nuclear expression significantly correlated with calpain 1 expression (*r* ^*2*^ = 0.568, *p* = <0.001 and *r* ^*2*^ = 0.504, *p* < 0.001, respectively). BMP7 cytoplasmic expression also significantly correlated with Calpastatin (*r* ^*2*^ = 0.253, *p* < 0.001), calpain‐2 (*r* ^*2*^ = 0.170, *p* = 0.001) and calpain‐4 (*r* ^*2*^ = 0.210, *p* < 0.001). Whilst BMP7 nuclear expression also significantly correlated with Calpastatin (*r* ^*2*^ = 0.103, *p* = 0.041) and calpain‐2 (*r* ^*2*^ = 0.128, *p* = 0.011), no correlation was observed with calpain‐4 (*r* ^*2*^ = 0.069, *p* = 0.0172).

### 3.6 BMP7 mRNA expression in the TCGA dataset

**FIGURE 3:**
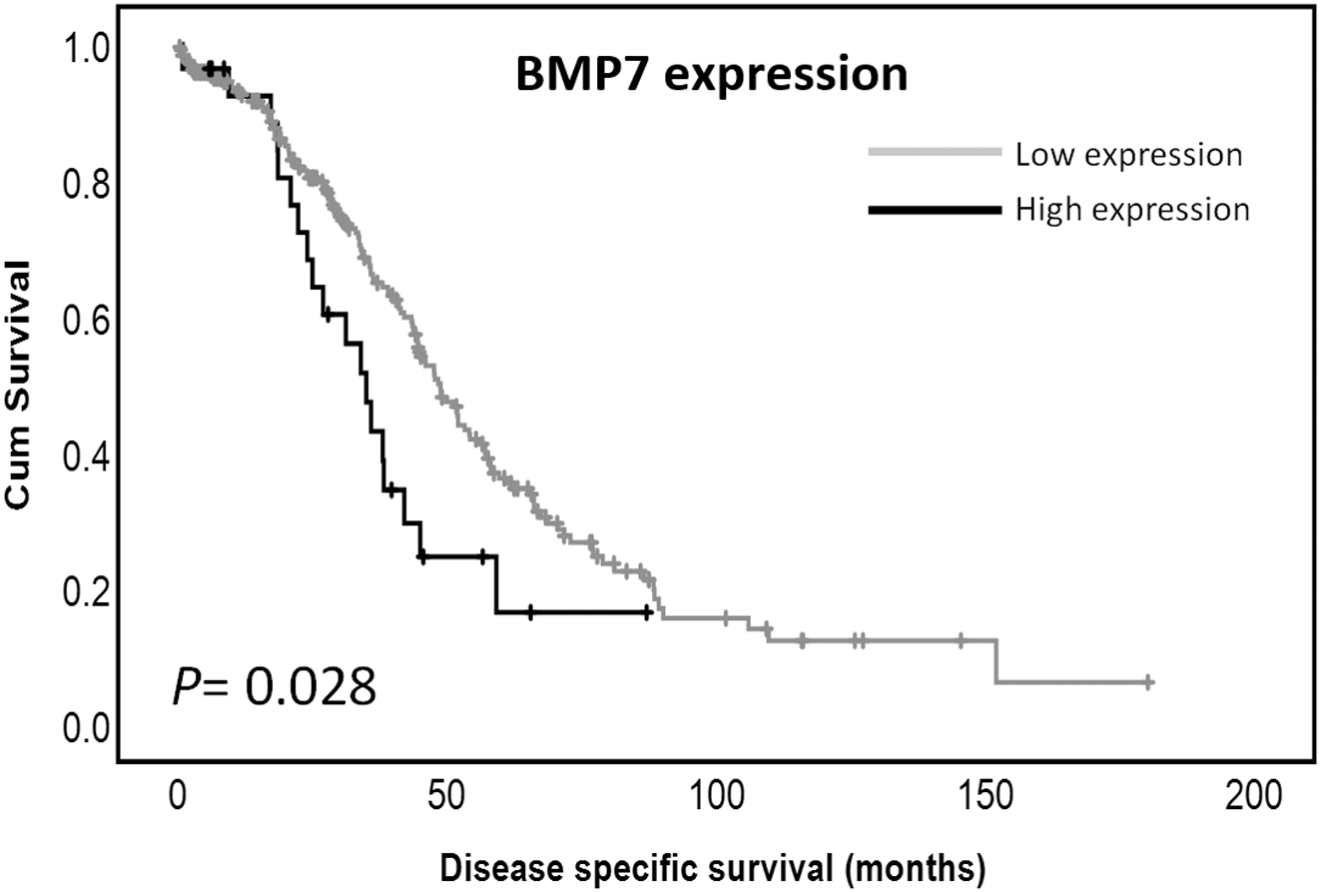
Kaplan–Meier survival curve of BMP7 mRNA expression and disease specific survival in the TCGA Pan Cancer Atlas cohort. Survival analysis shows significantly poor (*p* = 0.028) disease specific survival for ovarian cancer patients with tumours of high BMP7 expression compared to those with tumours of low BMP7 expression Significance was determined using the log‐rank test. High expression—black line, low expression—grey line.

High BMP7 mRNA expression was significantly associated with adverse disease specific survival (*p* = 0.028) (Figure 3) within the TCGA PanCancer Atlas cohort (*n* = 300). This is in line with the BMP7 protein expression data suggesting that high expression of BMP7 is associated with poor survival in ovarian cancer patients. BMP7 mRNA expression was not, however, associated with clinicopathological variables such as age at diagnosis (*χ* ^*2*^ = 3.287, d.f. = 1, *p* = 0.070) or histological grade (*χ* ^*2*^ = 1.004, d.f. = 3, *p* = 0.800). Associations with other clinicopathological factors could not be established due to limited availability of clinicopathological variables in this dataset.

## 4 DISCUSSION

Only a small number of studies have investigated the role of BMP7 in human carcinomas, including in prostate cancer, breast cancer, osteosarcomas and malignant melanomas.^28^, ^29^, ^30^, ^31^ However, BMP7 protein expression and its potential clinical significance in ovarian carcinomas has been even less well studied. A previous study, conducted by our group, suggested that BMP7 mRNA was highly differentially expressed in ovarian cancer cell lines derived after metastatic relapse (PEO4) compared to those derived from the primary cancer (PEO1).^18^ BMP7 mRNA expression had a 259.84‐fold increase in at 4 h and a 351.79‐fold increase at 24 h, suggesting a higher expression in more aggressive ovarian carcinomas than in early‐stage disease. Following on from such previous findings, this current study reports protein expression of BMP7 in a large cohort of human ovarian carcinomas. Expression was found in both the cytoplasm and nucleus of ovarian cancer cells and higher expression of both associated with several clinicopathological variables and overall survival of patients. Such findings are in line with our previous transcriptomic data, as high BMP7 protein expression was significantly associated with high FIGO stage, high tumour grade and an adverse overall survival in patient samples, suggesting a role in tumours of a more aggressive phenotype.

Data show that high BMP7 cytoplasmic and nuclear expression were each significantly associated with high FIGO stage suggesting a role in metastasis. By grouping cases into two categories—organ‐confined or spread data suggest that high cytoplasmic and nuclear BMP7 expression were significantly associated with tumours that had spread past the primary organ. It has been reported previously that elevated BMP7 expression was significantly associated with increased depth of tumour invasion, metastasis to liver, liver recurrence and advanced Dukes' classification (staging system used in colorectal cancer) in colorectal cancer.^32^ High BMP7 expression was reported to be significantly associated with greater nodal, lymphatic and venous invasion in gastric cancer.^33^ Similarly, in a recent study, knockdown of BMP7 in ovarian cancer cells A2780 cells inhibited migration and invasion.^34^ Such data suggests that BMP7 may play a significant role in metastasis and invasion in cancer and thus suggests the potential use of BMP7 as a biomarker for monitoring tumour progression at the molecular level.

High BMP7 cytoplasmic and nuclear expression were also significantly associated with high tumour grade. High grade tumour cells are substantially undifferentiated and exhibit nuclear pleomorphism with high mitotic counts. This association between BMP7 expression and high tumour grade suggests BMP7 plays a role in the development and progression of ovarian cancer by regulating cell proliferation. A study with serous ovarian cancer cells reported that activation of the BMP/SMAD‐5 signalling pathway resulted in enhanced proliferative activity.^35^ Alarmo and colleagues reported similar findings of BMP7 mRNA expression in breast cancer tissue as an increase in BMP7 gene copy number was significantly associated with high histological tumour grade and increased proliferation.^28^ Similarly, Guan et al. showed that downregulation in BMP7 activity led to reduced proliferation in ovarian cancer cells.^34^

High cytoplasmic and nuclear protein expression is associated with adverse overall survival. High expression of BMP7 mRNA was also significantly associated with poor disease specific survival in the TCGA PanCancer Atlas ovarian cancer cohort further strengthening the importance of BMP7 in ovarian cancer. BMP7 mRNA expression was not associated with the clinicopathological variables such patient age and histological grade in this cohort, and associations with other variables could not be established due to unavailability of data. However, the similarities between the two studies, with respect to patient survival strongly suggest that high BMP7 expression is of clinical importance in ovarian cancer patients. High BMP7 expression significantly associated with poorer survival in various cancers including hepatocellular carcinoma^36^ and oesophageal squamous cell carcinomas (ESCC).^37^ Kubiliute et al. reported that the DNA methylated status of the three gene panel of ADAMTS19, BMP7 and SFRP1 was predictive of a poorer overall survival in clear cell renal carcinoma.^38^ The relationship between BMP7 expression and overall survival in the current study suggests that BMP7 expression could potentially be used as a prognostic tool in ovarian cancer patients.

This significant association between high BMP7 expression and adverse overall survival is, however, to interpreted with caution as subsequent multivariate analyses showed that neither cytoplasmic nor nuclear BMP7 expression was an independent marker. Contrastingly, BMP7 was found to be an independent factor of overall survival in colorectal carcinoma^32^ and gastric carcinoma^33^ patients, however, this might be due to differences in sample size. BMP7 expression could, however, be an independent prognostic tool in certain subtypes of ovarian carcinoma and thus, if numbers were increased, it would be useful to conduct future univariate and multivariate analyses within the different histological subtypes.

No association was seen between cytoplasmic or nuclear BMP7 expression and progression‐free survival. Several reports, however, suggest that BMP7 may be important in progression‐free survival. BMP7 expression was associated with better outcomes post treatment in renal cell carcinomas^39^ as patients with positive BMP7 expression had higher progression‐free survival rates after treatment compared to those with negative expression. In addition, BMP7 expression in infiltrating urothelial carcinoma was associated with better progression‐free survival as high BMP7 expression was associated with a prolonged time to recurrence.^40^ Such studies suggest that BMP7 has a protective role in suppressing tumour growth and highlights the pleiotropic nature of BMP7 within different malignancies.

A significant association was found between high cytoplasmic and nuclear BMP7 expression and high‐grade serous carcinomas (HGSC). HGSCs are invasive tumours, characterized by highly undifferentiated cells and are the most aggressive of all ovarian carcinoma subtypes.^41^ Due to this, survival of patients with HGSC is significantly lower than in patients with low grade serous carcinomas.^42^ The involvement of BMP7 in the proliferation of tumours, disease progression and worse overall survival is therefore highlighted. Inversely, low cytoplasmic and nuclear BMP7 expression was associated with less aggressive disease. It has been proposed that each histological subtype is its own disease with its own aetiology and expression profiles.^13^

Whilst BMP7 expression was not associated with patient response to platinum‐based chemotherapy in the current cohort there is, however, accumulating evidence from reports on BMP7 expression suggesting that it is in other cancers. In oesophageal squamous cell carcinomas, BMP7 was expressed at significantly lower levels in patients who responded to chemotherapy compared to those who were chemo‐resistant.^43^ BMP7 expression was also found to correlate with the development of secondary drug resistance in mantle cell lymphoma. BMP7 expression was upregulated in patients who relapsed and developed secondary drug resistance, as seen in a gene profiling study.^44^ However, BMP signalling was seen to be upregulated in ovarian cancer cells treated with carboplatin, specifically by BMP2 secretion.^45^ The lack of association between BMP7 expression and chemotherapy response in this study, may indicate that the aggressiveness BMP7 provides in ovarian cancer may be EMT‐related. If not due to the evasion of therapy, the worse prognosis observed with high BMP7 expression in ovarian carcinomas may be due to the increased metastatic characteristics achieved during EMT, enabling rapid tumour progression.

Tumour cells with the ability to evade conventional cancer therapies remain in the body post treatment as residual disease. High cytoplasmic and nuclear BMP7 expression was significantly associated with residual tumours of greater size (>2 cm). Patients with residual disease are at an increased risk of subsequent tumour recurrence and thus, have a higher chance of cancer‐related mortality.^46^ In this regard, the association between BMP7 expression and residual disease also points towards the potential prognostic significance of BMP7 expression in ovarian cancer.

In vitro studies had previously suggested a potential link between the calpain system and BMP7. Strikingly, similar findings to the current study were reported previously when assessing calpain‐1 expression in the same cohort of ovarian carcinoma samples. High calpain‐1 expression was linked with the presence of residual disease and HGSC (as seen in high BMP7 expression) and low calpain‐1 expression was linked with low stage, no residual disease and clear cell, endometrioid and mucinous carcinomas. In addition, high calpain‐1 expression was associated with tumours that had metastasised from the primary organ.^18^ Both cytoplasmic and nuclear BMP7 expression were strongly correlated with calpain‐1 expression. The r‐squared value of 0.568 suggests a strong correlation according to the empirical classifications proposed by Cohen^27^ and suggests that the two proteins may have a joint role in ovarian cancer. Calpains, calcium dependent papain‐like enzymes, are cysteine proteases responsible for regulating a host of cellular processes such as signal transduction, cytoskeletal organization, cell survival and cell death.^47^, ^48^

Recent studies investigating the mechanisms of BMPs have elucidated the role of a certain class of molecules regarded as BMP antagonists.^13^ BMP antagonists have been receiving much attention due to their proposed role in cancer. In aggressive melanoma cells, BMP antagonist Noggin confers the cells with a resistance to BMP7^49^ and suppresses an EMT‐like transition of cells.^50^ In breast cancer cells, Noggin significantly inhibited osteolytic bone metastasis driven by the transcription factor FOXF2.^51^ Noggin appears to be directly opposing the action of BMP7 as it abrogates the protein's proposed function in facilitating EMT, metastasis and disease progression. In ovarian carcinomas, BMP antagonist Chordin, was found to enhance cell adhesions and reduce tumour motility to markedly suppress tumour progression.^52^ By blocking the BMP‐mediated signalling pathways, the antagonists exert an inhibitory biological effect and thus, are promising therapeutic agents in cancer treatment. Taken together, the data of this study suggests that BMP7 is important in metastasis and ovarian tumour progression, however the effect on biological mechanisms need to be investigated further both in vitro and in vivo. Employing BMP family antagonists as anti‐cancer drug developments could potentially improve the efficacy of ovarian cancer treatments. BMP antagonists may be used in combination with standard anti‐cancer drugs, to reduce disease progression and thus, improve the cytotoxic efficacy of standard anti‐cancer therapies.

## 5 CONCLUSIONS

The study provides evidence of the clinical importance of BMP7 in ovarian carcinomas. High expression of BMP7 is linked with disease progression and significantly associated with aggressive phenotypes including, advanced grade, FIGO stage, residual disease, and adverse overall survival. Larger independent studies are warranted.

## AUTHOR CONTRIBUTIONS

**Richa Vasan:** Formal analysis (supporting); methodology (supporting); writing – review and editing (lead). **Jahnavi Yadav:** Formal analysis (lead); investigation (equal); methodology (lead); writing – original draft (lead). **Radhika Aiyappa‐Maudsley:** Formal analysis (supporting); methodology (supporting). **Suha Deen:** Methodology (supporting); resources (lead). **Sarah J Storr:** Formal analysis (supporting); methodology (supporting). **Stewart G Martin:** Conceptualization (lead); formal analysis (supporting); funding acquisition (lead); project administration (lead); supervision (lead); writing – review and editing (supporting).

## CONFLICT OF INTEREST STATEMENT

The authors confirm that there are no conflicts of interest.

## Supporting information


**
**Figure** **S1** .** Western blot confirming anti‐BMP7 specificity in ovarian cancer cells SKOV3, A2780 and OVCA433, and breast cancer cells T47D and MCF7. A single thick band was visualized for these cell lines.at the correct size 49 kDa. A few faint bands for BMP7 protein aggregates were also observed.
Supplementary table 1. Patient clinicopathological variables
Supplementary table 2. Multivariate (Cox proportional hazard regression) analysis of overall survival

## Data Availability

Anonymised data can be made available by approaching the corresponding author.
