# Supplementary material for: High BMP7 expression is associated with poor prognosis in ovarian cancer
Source: J Cell Mol Med. 2023 Sep 8;27(21):3378–87. doi: 10.1111/jcmm.17951 (PMC10623526; doi:10.1111/jcmm.17951)
Supplement: Supplementary file 1 — Figure S1 . Western blot confirming anti‐BMP7 specificity in ovarian cancer cells SKOV3, A2780 and OVCA433, and breast cancer cells T47D and MCF7. A single thick band was visualized for these cell lines.at the correct size 49 kDa. A few faint bands for BMP7 protein aggregates were also observed. Supplementary table 1. Patient clinicopathological variables Supplementary table 2. Multivariate (Cox proportional hazard regression) analysis of overall survival [file JCMM-27-3378-s001.docx]

**Supplementary data**

**Supplementary figure 1**. Western blot confirming anti-BMP7 specificity in ovarian cancer cells SKOV3, A2780 and OVCA433, and breast cancer cells T47D and MCF7. A single thick band was visualised for these cell lines.at the correct size 49 kDa. A few faint bands for BMP7 protein aggregates were also observed.

**Supplementary table 1. Patient clinicopathological variables**

| **Variables** | **Number of Patients** | **Percentage (%)** |
| --- | --- | --- |
| **Histological Subtypes** |  |  |
| High grade serous | 337 | 59.7 |
| Mucinous | 60 | 10.6 |
| Endometrioid | 68 | 12.1 |
| Clear cell | 53 | 9.4 |
| Low grade serous | 30 | 5.3 |
| Borderline serous | 15 | 2.7 |
| Borderline mucinous | 1 | 0.2 |
| **Age** |  |  |
| ≤61 | 295 | 52.4 |
| ≥61 | 268 | 47.6 |
| **Grade** |  |  |
| 1 | 48 | 8.5 |
| 2 | 90 | 16 |
| 3 | 425 | 75.5 |
| **Stage** |  |  |
| 1 | 203 | 36.7 |
| 2 | 64 | 11.6 |
| 3 | 345 | 44.3 |
| 4 | 41 | 7.4 |
| **Adjuvant therapy** |  |  |
| Platinum-based chemotherapy | 357 | 63.3 |
| Non-platinum-based chemotherapy | 6 | 1.1 |
| No chemotherapy | 80 | 14.2 |
| No information | 121 | 21.5 |
| **Response to chemotherapy** |  |  |
| Refractory and resistance | 66 | 17.7 |
| Sensitivity | 307 | 82.3 |
| **Residual disease** |  |  |
| No residual tumour | 311 | 62.2 |
| Residual tumour<2 cm | 58 | 11.6 |
| Residual tumour>2 cm | 131 | 26.2 |
| **Progression status** |  |  |
| No recurrence | 137 | 32.9 |
| Recurred | 280 | 67.1 |
| **Survival status** |  |  |
| Living | 234 | 42 |
| Deceased | 323 | 58 |

**Supplementary table 2. Multivariate (Cox proportional hazard regression) analysis of overall survival**

|  |  |  |  |  | **95% confidence Interval for Exp (B)** | |
| --- | --- | --- | --- | --- | --- | --- |
| **Variables** | | | **P value** | **Hazard ratio** |  |  |
|  |  |  |  |  | **Lower** | **Upper** |
| **Multivariate (overall survival)** | | |  |  |  |  |
| Histological subtypes | | | 0.936 | 0.994 | 0.854 | 1.156 |
| Age (median 61) | | | 0.469 | 1.139 | 0.8 | 1.622 |
| Grade | | | 0.051 | 1.1701 | 0.997 | 2.902 |
| FIGO stage | | | **0.002** | 1.456 | 1.155 | 1.837 |
| Platinum sensitivity | | | **<0.001** | 2.981 | 1.934 | 4.596 |
| Residual disease | | | 0.067 | 1.236 | 0.985 | 1.552 |
| Cytoplasmic BMP7 expression | | | 0.327 | 1.202 | 0.832 | 1.737 |
| **Multivariate (overall survival)** | | |  |  |  |  |
| Histological subtypes | | | 0.755 | 0.976 | 0.838 | 1.137 |
| Age (median 61) | | | 0.419 | 1.157 | 0.812 | 1.65 |
| Grade | | | 0.062 | 1.658 | 0.976 | 2.819 |
| FIGO stage | | | **0.002** | 1.451 | 1.151 | 1.829 |
| Platinum sensitivity | | | **<0.001** | 2.855 | 1.864 | 4.374 |
| Residual disease | | | 0.056 | 1.25 | 0.994 | 1.571 |
| Nuclear BMP7 expression | | | 0.909 | 1.02 | 0.722 | 1.441 |

Significant results (P<0.05) are highlighted in bold.
